# *Staphylococcus aureus* Lipoteichoic Acid Inhibits Platelet Activation and Thrombus Formation via the Paf Receptor

**DOI:** 10.1093/infdis/jit398

**Published:** 2013-08-02

**Authors:** Amie K. Waller, Tanya Sage, Christopher Kumar, Thomas Carr, Jonathan M. Gibbins, Simon R. Clarke

**Affiliations:** 1 School of Biological Sciences,; 2 Institute for Cardiovascular and Metabolic Research, University of Reading, Whiteknights, United Kingdom

**Keywords:** *Staphylococcus aureus*, platelets, lipoteichoic acid

## Abstract

Impaired healing is common in wounds infected with the major human pathogen *Staphylococcus aureus*, although the underlying mechanisms are poorly understood. Here, we show that *S. aureus* lipoteichoic acid (LTA) inhibits platelet aggregation caused by physiological agonists and *S. aureus* and reduced platelet thrombus formation in vitro. The presence of D-alanine on LTA is necessary for the full inhibitory effect. Inhibition of aggregation was blocked using a monoclonal anti-platelet activating factor receptor (PafR) antibody and Ginkgolide B, a well-defined PafR antagonist, demonstrating that the LTA inhibitory signal occurs via PafR. Using a cyclic AMP (cAMP) assay and a Western blot for phosphorylated VASP, we determined that cAMP levels increase upon platelet incubation with LTA, an effect which inhibits platelet activation. This was blocked when platelets were preincubated with Ginkgolide B. Furthermore, LTA reduced hemostasis in a mouse tail-bleed assay.

*Staphylococcus aureus* is an important opportunistic human pathogen and the cause of a large burden of morbidity and mortality. The ability of the pathogen to bind to and activate platelets, small (2–4 µm), blood cells responsible for maintaining normal hemostasis leads to the formation of platelet-bacteria thrombi on the surface of heart valves, which is required for the development of endocarditis since platelets attached to damaged valves serve as foci for attachment of bacteria circulating in the blood [1]. Several studies have shown that *S. aureus* binds platelets and induces their aggregation. The pathogen possesses a variety of surface proteins known as microbial surface components reacting with adhesive matrix molecules (MSCRAMMs), some of which are virulence factors in models of *S. aureus* endocarditis. MSCRAMMs attach the bacterium to platelets, either indirectly, by binding to fibrinogen, simultaneously bound to the platelet surface by integrin αIIbβ3, or by binding directly to αIIbβ3, thus inducing outside-in signaling and platelet activation [2, 3]. Such observations rely on washed *S. aureus* cells and thus ignore the contribution of bacterial molecules secreted into the extracellular milieu.

Although induction of thrombus formation by *S. aureus* has been characterized extensively, infection of wounds by this pathogen frequently results in impaired healing, the mechanisms of which are not fully understood [4]. *S. aureus* extracellular proteins Efb inhibits platelet aggregation by binding to fibrinogen [5]. Inhibition of platelet activity by Efb or pharmacological antagonists causes decreased killing of *S. aureus* in whole blood and increases the lethality of *S. aureus* infection in a mouse model [6].

*S. aureus* lipoteichoic acid (LTA) was previously shown to inhibit activation of platelets, although a role in hemostasis its relevance to the *S. aureus*-platelet interaction and the mechanism(s) by which inhibition is achieved are not understood [7]. In this study, LTA inhibited activation of human platelets by physiological agonists and *S. aureus*. Furthermore, LTA inhibits platelet function and thrombus formation in vivo by binding platelet activating factor receptor (PafR), a phospholipid receptor that binds LTA and is associated with an increased platelet intracellular cyclic adenosine monophosphate (cAMP) concentration.

## METHODS

### Reagents

Collagen was obtained from Nycomed, thrombin, Ginkoglide B, Mouse immunoglobulin G (IgG) from Sigma, and cross-linked collagen-related peptide (CRP-XL) from R. Farndale (University of Cambridge, UK). Anti-LTA (pagibaximab) was provided by Biosynexus Inc. Anti-TLR2 (T2.5) was purchased from eBioscience, anti-CD14 (UCH-M1) from AbD serotec, anti-PafR from Cayman Chemical, anti-PhosphoVASP from Cell Signaling Technology.

### Bacterial Strains Used

Wild-type *S. aureus* SA113 was used with *S. aureus* SA113 Δ*dltABCD* [8], *S. aureus* SA113 Δ*tagO* [9], and *S. aureus* SA113 Δ*lgt* [10]. *S. aureus* SEJ1 and isogenic strains Δ*gdpP*, Δ*gdpP*Δ*ltaS*, and pCN34-*ltaS* were used for mutant studies [11]. *Bacillus subtilis* 128 and *Streptococcus pneumoniae* D34 were used.

### LTA Extraction

*S. aureus* was grown in BHI 37°C and centrifuged at 20 463 g for 15 minutes. The pellet was resuspended in 50% butanol/water. LTA was resuspended in a 1 mM sodium acetate, 15% 1-propanol buffer followed by a 15%–60% 1-propanol elution gradient, dialyzed against dH_2_0, and the concentration determined by phosphate assay [12].

### LTA From *S. aureus* Culture Supernatants

*S. aureus* cultures were centrifuged at *c.* 12 000 g for 10 minutes to remove cells. In total, 2.3M (NH_4_)_2_SO_4_ was added to the supernant overnight at 4°C. The supernatant was centrifuged at 20 000 g for 20 minutes at 4°C and the pellet resuspended in 2 mL of phosphate-buffered saline (PBS). To standardize supernatant preparations, including those lacking LTA, proteins carried over were quantified by Bradford assay. Where appropriate, LTA concentrations were determined as above.

### Preparation of Human Platelets

Human blood was obtained from healthy volunteers who gave informed consent. Ethical approval was obtained from the University of Reading Research Ethics Committee. Platelets were prepared as described elsewhere [13]. In total, 4 × 10^8^ platelets/mL were incubated for 15 minutes with LTA and stimulated by agonists. Aggregation was measured in an optical aggregometer (Chronolog). Percentage inhibition of aggregation by LTA was calculated by dividing maximal aggregation of LTA-treated samples by the aggregation achieved by the given agonist alone. Centrifuged *S. aureus* were washed 3 times in Tyrodes buffer and adjusted for a final experimental OD_600_ 0.3. Aggregation was measured up to 15 minutes.

### Flow Cytometry

In total, 5 µL of platelet-rich plasma (PRP; 4 × 10^8^ cells/mL) was incubated with anti-PafR (50 µg/mL), IgG2a (50 µg/mL), or Tyrode buffer for 30 minutes, then incubated with various concentrations of FITC-LTA for 15 minutes. Fluorescence intensity of the sample was measured using a BD Accuri C6 flow cytometry; 10 000 events per sample were measured.

### Measurement of Intracellular [Ca^2+^]_i_

Platelets were preloaded with the fluorescent dye Fluo-4NW as described elsewhere [13]. PRP was preincubated with LTA for 15 minutes before being stimulated with CRP-XL, and calcium release was measured using a Fluoroskan reader (Thermolab Systems) at 485/530 nm.

### In Vitro Thrombus Formation

Whole citrated blood was perfused through a Vena8Biochip (Cellex, Dublin). Z-stack images were taken every 30 seconds using a Nikon eclipse (TE2000-U) microscope; data were analyzed using Slidebook5 software (Intelligent Imaging Innovations, Denver, USA)

### Tail-bleeding Assay

Procedures were approved by the University of Reading Animal Ethics Committee and the Home Office. Sixteen age-matched C57BL/6 mice were killed using ketamine (80 mg/kg) and xylazine (5 mg/kg) administered intraperitoneally prior to a tail biopsy. Time to cessation of bleeding was measured up to 20 minutes.

## RESULTS

### *S. aureus* LTA Inhibits Platelets Aggregation by Various Platelet Agonists

**Figure 1.**
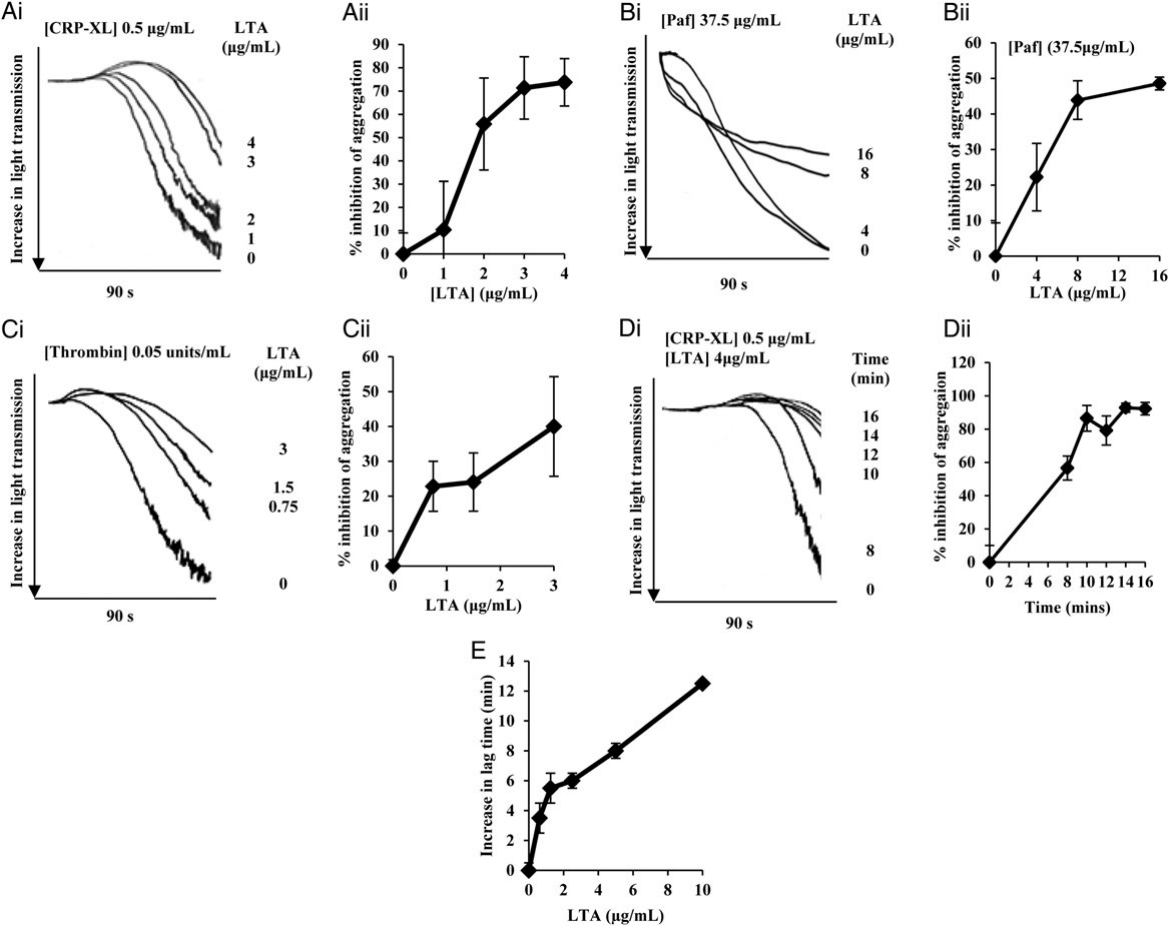
LTA from *Staphylococcus aureus* inhibits platelet aggregation. Washed human platelets (4 × 10^8^ cells/mL) or PRP were preincubated with LTA or tyrodes buffer and stimulated with various platelet agonists. Aggregation was measured as change in light transmission. *Ai*–*Di*, Representative aggregation traces of platelets incubated with LTA and stimulated with various platelet agonists. All aggregation traces commence upon addition of agonist. *Aii*–*Dii*, Data are plotted as percentage inhibition of aggregation or percentage aggregation (vehicle treated representing 100% aggregation) and represent mean values ± SEM. *A*, Platelets were pre-incubated with LTA at a range of concentrations followed by stimulation with CRP-XL (0.5 µg/mL). Aggregation was measured for 90 seconds. *B*, Platelets were preincubated with LTA at a range of concentrations followed by stimulation with Paf (37.5 µg/mL). Aggregation was measured for 90 seconds. *C*, Platelets were pre-incubated with LTA at a range of concentrations followed by stimulation with thrombin (0.05 units/mL). Aggregation was measured for 90 seconds. *D*, Platelets were preincubated with LTA (4 µg/mL) for 8, 10, 12, 14, and 16 minutes followed by stimulation with CRP-XL (0.5 µg/mL). Aggregation was measured for 90 seconds. *E*, PRP was pre-incubated with various concentrations of LTA for 15 minutes followed by stimulation with whole *S. aureus* SA113 cells (2 × 10^8^ cells/mL). Aggregation was measured for up to 20 minutes. Data are plotted as increase in lag time and represent mean values ± SEM. *P < .05 Abbreviations: CRP-XL, cross-linked collagen-related peptide; LTA, lipoteichoic acid; PRP, platelet-rich plasma; SEM, standard error of the mean.

LTA is found at high concentrations within *S. aureus* cells and the extracellular milieu [14] and could thus interact with platelets during infection. LTA present in the supernatant and *S. aureus* cell envelope have the same structure [15]. In this study we investigated LTA inhibition of platelet aggregation. First, we confirmed equal inhibitory activity for LTA purified from the supernatant and cells (Supplementary Figure 1*A*). For efficiency, remaining experiments used LTA extracted from the cell envelope. To determine which signaling pathways LTA inhibits, aggregation assays were performed with well-characterized platelet agonists. Purified LTA from *S. aureus* SA113 was preincubated with washed human platelets before activation with CRP-XL, a collagen receptor Glycoprotein VI selective agonist (Figure 1*Ai* and 1*Aii*), platelet activating factor (paf; Figure 1*Bi* and 1*Bii*) or thrombin (Figure 1*Ci* and 1*Cii*). LTA inhibited aggregation in a dose-dependent manner with all agonists. LTA was incubated for varying periods of time with washed platelets to observe any time-dependent effects on aggregation. Using 4 µg/mL LTA, inhibition of aggregation increased in a time-dependent manner (Figure 1*Di* and 1*Dii*). Platelet activation was inhibited over extended time periods (Supplementary Figure 1*B*). The highest concentration of LTA used with thrombin (3 µg/mL), CRP-XL (4 µg/mL), and paf (16 µg/mL) as platelet agonists produced a 40%, 85%, and 50% reduction in platelet aggregation respectively, thus showing LTA to be a potent inhibitor of platelet aggregation.

Whole bacterial cells stimulate platelet activation via formation of fibrinogen or fibronectin bridges between integrin αIIbβ3 and *S. aureus* MSCRAMMs [3, 16, 17]. Having demonstrated LTA inhibition of platelet activation by physiological agonists, we examined the ability of exogenous LTA to inhibit *S. aureus* induced platelet aggregation in PRP (Figure 1*E*). Increasing LTA concentrations increased the lag time to platelet aggregation.

**Figure 2.**
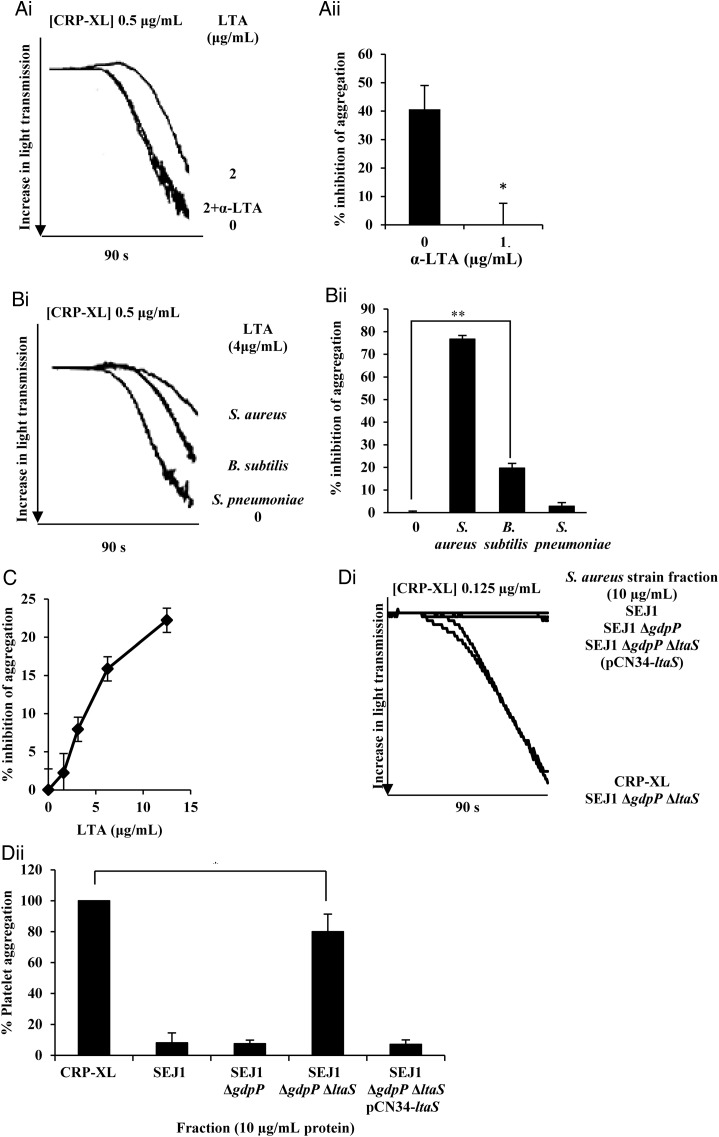
The inhibition is due to the presence of *Staphylococcus aureus* LTA. *A*, Washed human platelets (4 × 10^8^ cells/mL) were preincubated with anti-LTA antibody (1 µg/mL) before incubation for 15 minutes with LTA (2 µg/mL) followed by stimulation with CRP-XL (0.5 µg/mL). Aggregation was measured as change in light transmission for 90 seconds. NB: In Ai, lines representing platelets treated with 2 µg/mL + anti-LTA antibodies and untreated platelets, overlap extensively. *B*, Washed platelets were incubated for 15 minutes with LTA extracted from *S. aureus* SA113 (4 µg/mL)*, Bacillus subtilis,* or *Streptococcus pnuemoniae* D34 (12.5 µg/mL) followed by stimulation with CRP-XL (0.5 µg/mL). *C*, Washed platelets were incubated for 15 minutes with LTA extracted from *B. subtilis* at a range of concentrations followed by stimulation with CRP-XL (0.5 µg/mL). *D*, Washed platelets were incubated for 15 minutes with supernatant from *S. aureus* SEJ1, SEJ1 Δ*gdpP*, SEJ1 Δ*ltaS* Δ*gdpP* or SEJ1 Δ*ltaS* Δ*gdpP* pCN34-*ltaS* (10 µg/mL), followed by stimulation with CRP-XL (0.5 µg/mL). (*Ai*, *Bi*, *Di*) Representative aggregation traces of washed platelets. Aggregation was measured for 90 seconds. (*Aii*, *Bii*, *C*, *Dii*) Data are plotted as percentage inhibition of aggregation represent mean values ± SEM. *P < .05, **P < .001. Abbreviations: CRP-XL, cross-linked collagen-related peptide; LTA, lipoteichoic acid; SEM, standard error of the mean.

To confirm that the observed inhibition was due to LTA rather than a copurifying contaminant, platelets were pretreated with monoclonal anti-LTA and LTA before stimulation with CRP-XL. This blocked the LTA inhibitory effect (Figures 2*Ai* and 2*Aii*). An isotype (IgG1) matched control had no effect (results not shown). Lipoproteins that can copurify with LTA are sometimes responsible for the immunological activities that have been assigned to LTA [18]. *S. aureus* wall teichoic acid (WTA) has a similar structure to LTA. LTA was extracted from SA113 Δ*tagO* and SA113 Δ*lgt*, which lack WTA and lipoproteins, respectively, and tested in the same manner. LTA extracted from both of these strains inhibited platelets to the same levels as LTA from SA113 (Supplementary Figure 2), confirming LTA platelet inhibitory activity and excluding any effect from lipoproteins or WTA.

To determine whether this inhibition was restricted to *S. aureus* LTA, the molecule was extracted and purified from 2 other Gram-positive bacteria and tested for its ability to inhibit platelet aggregation. *Bacillus subtilis* is a nonpathogenic species that, like *S. aureus*, produces LTA with a 1,3-linked polyglycerolphosphate chain tethered to the membrane by a diglucosyl-diacylglycerol glycolipid. Glycerolphosphate subunits are esterified with D-alanine [19]. *Streptococcus pneumoniae* LTA consists of a repeating ribitol-galactose backbone with phosphocholine and D-alanine residues attached [20]. *S. pneumoniae* LTA showed no inhibition, whereas *B. subtilis* LTA caused a dose-dependent inhibition up to a maximum of 20% (Figure 2*Bi*, 2*Bii*, and 2*C*); we were unable to solubilize high enough concentrations of *B. subtilis* LTA to achieve saturation.

### The Supernatant of an *S. aureus* Mutant Which Lacks LTA Is Unable to Inhibit Platelet Activation

*S. aureus* mutant strains lacking LTA only grow under osmotically stabilizing conditions or by acquiring compensatory mutations [11]. *S. aureus* SEJ1 (RN4220 *spa*) was used to construct an LTA-deficient (Δ*ltaS*) strain*,* but in order for the Δ*ltaS* to be viable, *gdpP* must also be mutated [11]. Cells of the parental SEJ1 and isogenic strains Δ*gdpP*, Δ*gdpP*Δ*ltaS*, and Δ*gdpP*Δ*ltaS* containing pCN34-*ltaS*, a complementation plasmid expressing *ltaS* from its native promoter, were grown and OD_600_ of *c*. 0.5 and LTA in the supernatant was precipitated using (NH_4_)_2_SO_4_. To ensure that consistent amounts of material were used, the amount of protein precipitated along with the LTA was determined by Bradford assay to standardize the preparations. As expected, only supernatant from the Δ*gdpP*Δ*ltaS* lacked LTA upon Western blotting (results not shown). In each experiment, precipitate was used to a final concentration of 10 μg/mL of exoprotein. Supernatant from all strains, except Δ*gdpP*Δ*ltaS*, inhibited platelet activation (Figure 2*Di* and 2*Dii*).

We were unable to assess the ability of these stains to induce platelet aggregation, as *S. aureus* RN4220 proved unable to induce aggregation despite incubations of up to 1 hour (results not shown).

### LTA Produced From *S. aureus* Δ*dltABCD* Has a Reduced Ability to Inhibit Platelet Aggregation

**Figure 3.**
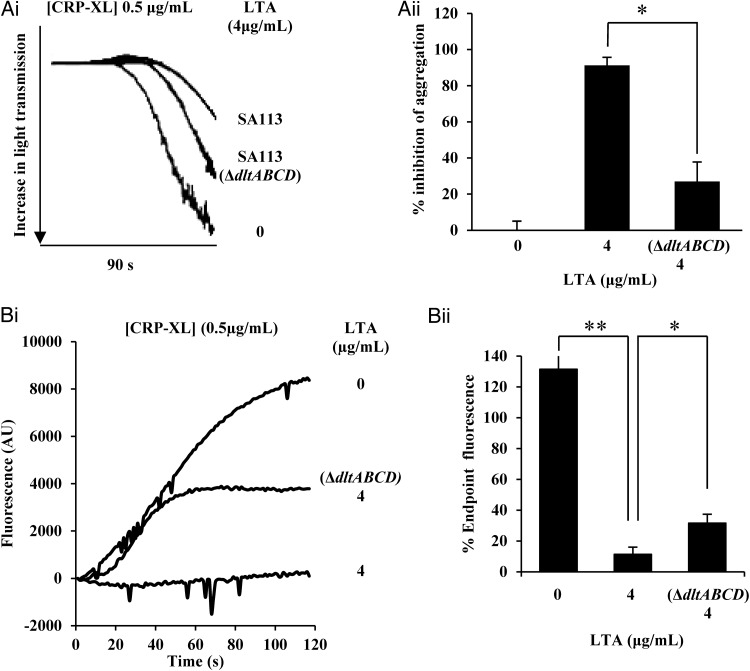
LTA from *Staphylococcus aureus* Δ*dltABCD* has a reduced ability to inhibit platelet aggregation. *A*, Washed human platelets (4 × 10^8^ cells/mL) were incubated for 15 minutes with LTA extracted from *S. aureus* strains SA113 or SA113 Δ*dltABCD* (4 µg/mL) followed by stimulation with CRP-XL (0.5 µg/mL). *B*, Platelets in PRP were preloaded with Fluo-4NW dye. Platelets were then pre-incubated with LTA extracted from *S. aureus* strains SA113 or SA113 Δ*dltABCD* (4 µg/mL) or Tyrodes buffer for 15 minutes followed by stimulation by CRP-XL (0.5 µg/mL). Intracellular mobilization of calcium was measured by spectrofluorimetry for 120 seconds. *Ai*, Representative aggregation traces of washed platelets. Aggregation was measured for 90 seconds. *Ai*, Representative aggregation trace. *Aii*, Data are plotted as percentage maximum fluorescence (vehicle treated representing 100% aggregation) and represent mean values ± SEM. *Bi*, Representative calcium flux trace. *Bii*, Data are plotted as percentage endpoint fluorescence (vehicle treated representing 100% aggregation) and represent mean values ± SEM. *<.01, **P < .005. Abbreviations: CRP-XL, cross-linked collagen-related peptide; LTA, lipoteichoic acid; SEM, standard error of the mean.

D-alanine residues are important for various functions of LTA in different biological systems. In *S. aureus* the addition of D-alanine to the LTA chain is encoded by the *dlt* operon [8]. LTA was extracted and purified from *S. aureus* SA113 Δ*dltABCD* and preincubated with washed human platelets. LTA purified from *S. aureus* SA113 Δ*dltABCD* showed a significant reduction (*c*. 60%) in its ability to inhibit the platelet aggregation (Figure 3*Ai*), compared to LTA from the parental wild-type strain (Figure 3*Aii*). Activation of platelets by whole *S. aureus* Δ*dltABCD* was indistinguishable from the parent (results not shown); thus it appears that LTA needs to be released from *S. aureus* to exert its inhibitory effect.

### LTA Inhibits [Ca2^+^] Mobilization

During the initial stages of platelet activation, intracellular calcium stores are mobilized to modulate downstream signaling. In order to further characterize the effect of LTA on platelets during the early stages of aggregation, an assay to determine intracellular calcium mobilization and influx was performed. Platelets preincubated with LTA showed a significantly reduced ability to mobilize calcium when challenged with CRP-XL compared to a vehicle treated control (*P* < .001; Figure 3*Bi*). Over the course of the assay this equated to a 70% reduction in intracellular calcium levels (Figure 3*Bii*). Because of its reduced inhibitory potency, calcium release stimulated by LTA from SA113 Δ*dltABCD* was assayed. From the mean trace of fluorescence over the course of the experiment a repeatable difference was observed (Figure 3*Bi*). The peak end point fluorescence caused by LTA derived from wild-type and Δ*dltABCD* strains were significantly different (Figure 3*Bii*).

### LTA Inhibition of Platelet Activation Can Be Blocked Using Anti-Paf Receptor Antibodies and Ginkgolide B

**Figure 4.**
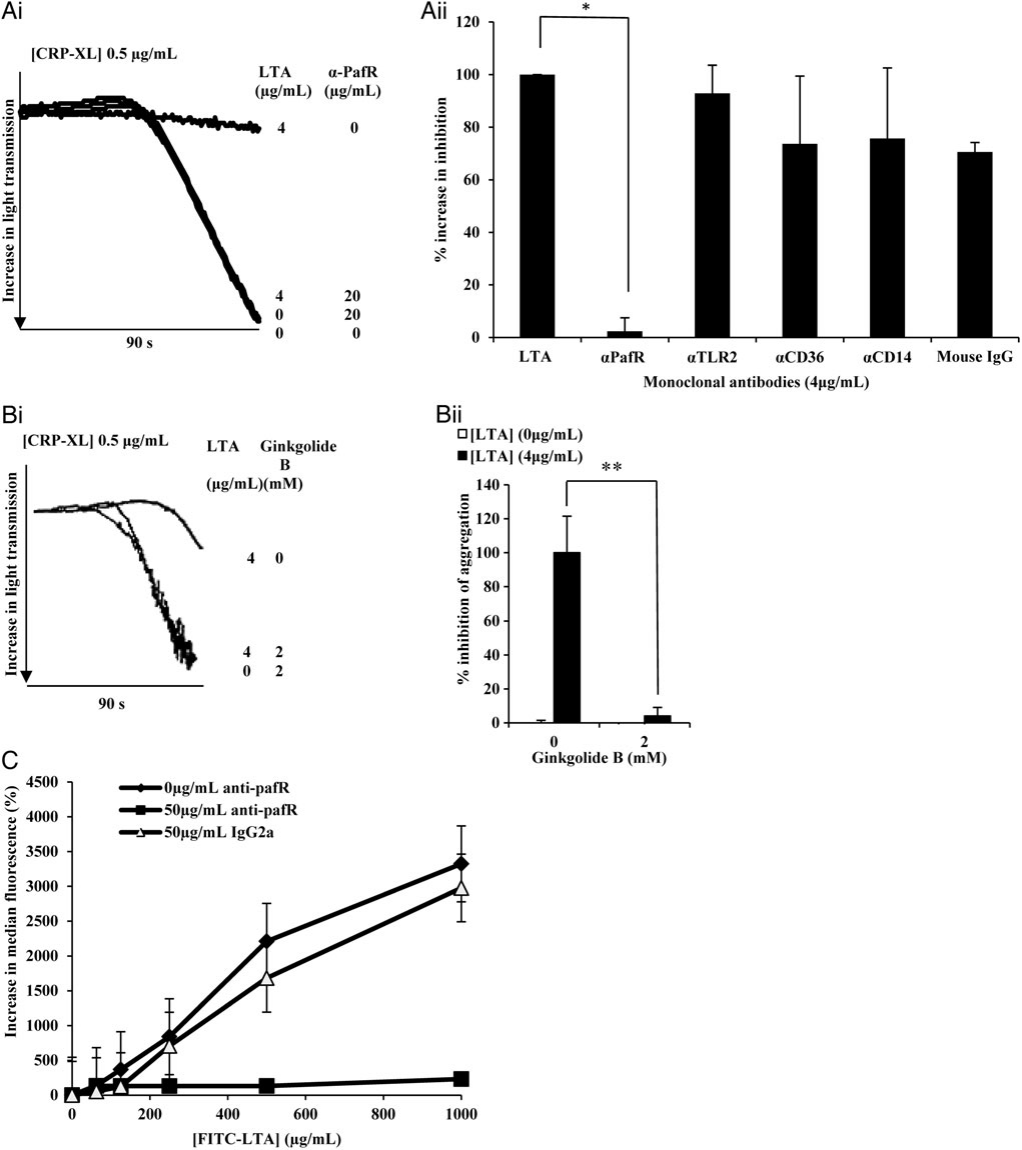
LTA acts through PafR to inhibit platelets. *A*, Washed platelets were incubated with anti-PafR (4 µg/mL), anti-TLR2 (4 µg/mL), anti-CD14 (4 µg/mL), Mouse IgG (4 µg/mL) or Tyrodes buffer for 30 minutes before addition of LTA (4 µg/mL) for 15 minutes. *B*, Washed platelets were incubated with Ginkgolide B (2 mM) or Tyrodes buffer for 30 minutes before the addition of LTA (4 µg/mL) for 15 minutes. Platelets were then stimulated with CRP-XL (0.5 µg/mL). NB: In *Bi*, lines representing platelets treated with 4 µg/mL LTA + 2 µM Ginkgolide B and 0 µg mL^−1^ LTA + 2 µM Ginkgolide B, overlap extensively. *A* and *B*, Platelet aggregation was measured as change in light transmission and recorded for 90 seconds. Data are plotted as percentage inhibition of aggregation (normalized so that LTA treatment represents 100% inhibition) and represent mean values ± SEM. *P < .0001, **P < .01. *C*, PRP (4 × 10^8^ cells/mL) was incubated for 30 minutes with anti-PafR (50 µg/mL), IgG2_a_ (50 µg/mL) or Tyrode buffer before the addition of FITC-LTA at several concentrations for 15 minutes. Samples were run through a BD Accuri C6 flow cytometer and median fluorescence was recorded. Data are plotted as percentage median increase in fluorescence when compared to a Tyrode buffer only control and represent mean values ± SEM. Abbreviations: CRP-XL, cross-linked collagen-related peptide; IgG, immunoglobulin G; LTA, lipoteichoic acid; PafR, platelet activating factor receptor; SEM, standard error of the mean.

In different cell types, LTA has previously been reported to bind 4 receptors CD14 [21], CD36 [22], TLR2 [23], and platelet activating factor receptor (PafR) [24]. Monoclonal antibodies with blocking activity for TLR2 and CD14, along with anti-PafR and –CD36 monoclonal antibodies were each tested for their ability to block LTA inhibition (Figure 4*Ai*). The anti-CD14 and –PafR antibodies were isotype matched. No significant blocking of LTA inhibition occurred with the anti-CD14, -CD36, -TLR2, or mouse IgG (negative control). However anti-PafR abolished LTA-mediated inhibition (Figure 4*Ai* and 4*Aii*). Furthermore, ginkgolide B, a specific PafR antagonist [25] blocked LTA-mediated platelet inhibition (Figure 4*Bi*), reducing it to 0% inhibition (Figure 4*Bii*). These data demonstrate a role for PafR on platelets as an LTA receptor.

Using flow cytometry, anti-PafR antibody abolished binding of FITC labelled LTA to platelets, demonstrating that PafR is the only receptor for the molecule (Figure 4*C*).

We tested whether anti-PafR antibody blocking was due to a nonspecific interaction with LTA, rather than blocking of PafR. We were unable to deplete samples of their inhibitory effect using anti-PafR antibodies, linked to protein A coated magnetic beads (results not shown). The blocking effect of anti-PafR was not due to cross-reactivity with LTA.

### LTA Causes Raised cAMP Levels to Cause Inhibition of Platelet Activation

**Figure 5.**
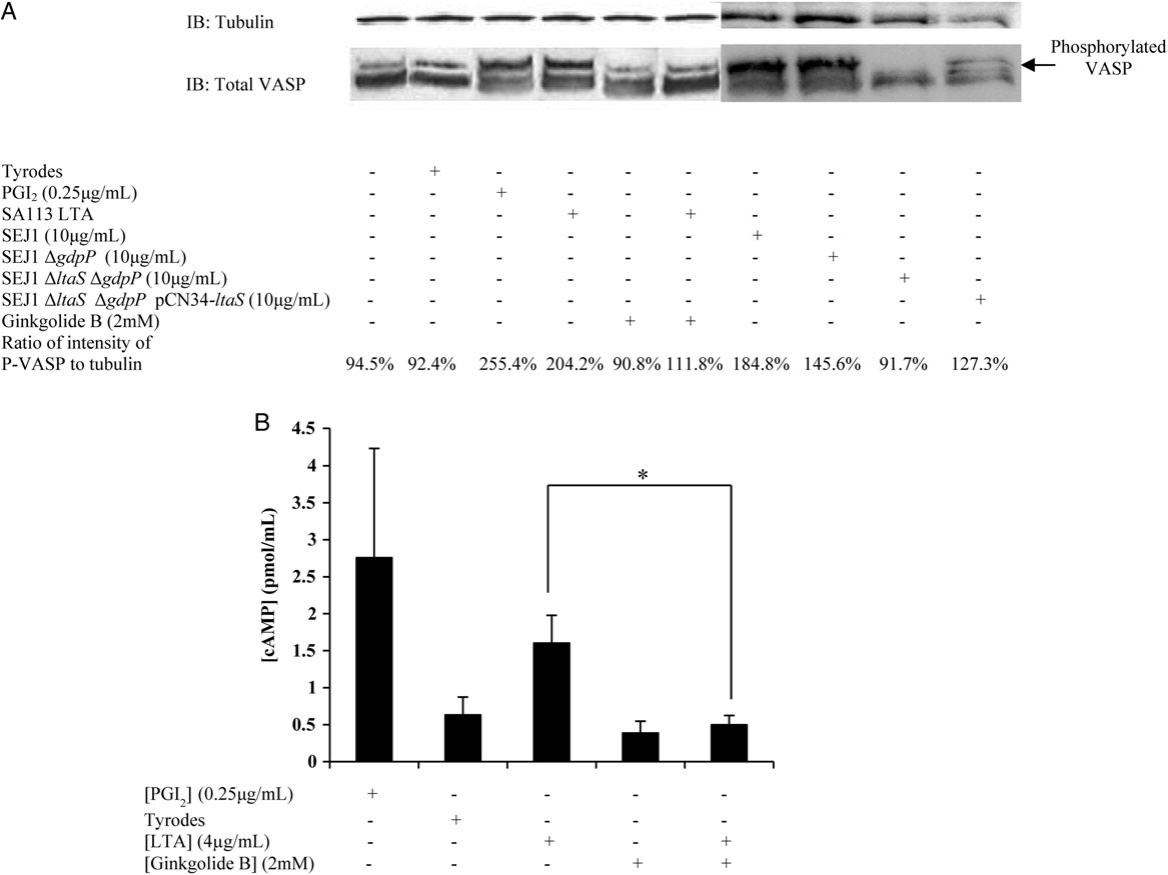
Incubation of platelets with LTA increases cAMP concentrations. *A*, Platelets (8 × 10^8^ cells/mL) were pretreated with either Ginkgolide B (2 mM) or tyrodes buffer for 30 minutes. Platelets were then treated with LTA from *Staphylococcus aureus* SA113 (4 µg/mL), PGI_2_ (0.25 µg/mL), supernatant from *S. aureus* SEJ1, SEJ1 Δ*gdpP*, SEJ1 Δ*ltaS* Δ*gdpP* or SEJ1 Δ*ltaS* Δ*gdpP* pCN34-*ltaS* (10 µg/mL), or Tyrodes buffer. Lysates were immunoblotted with an anti-VASP antibody. *B*, Platelets (8 × 10^8^ cells/mL) were pretreated with either Ginkgolide B (2 mM) or tyrodes buffer for 30 minutes. Platelets were then treated with LTA (4 µg/mL), PGI_2_ (0.25 µg/mL), or tyrodes buffer. Samples were then assayed for cAMP concentration determined by ELISA. Data represent mean values ± SEM. *P < .05. Abbreviations: cAMP, cyclic adenosine monophosphate; ELISA, enzyme-linked immunosorbent assay; LTA, lipoteichoic acid; SEM, standard error of the mean; VASP, vasodilator-stimulated phosphoprotein.

Our finding that LTA inhibited Ca^2+^ flux in platelets led us to hypothesize that an increase of cAMP would occur in platelets upon incubation with LTA, which could be blocked with ginkgolide B. Increased cAMP concentrations attenuate Ca^2+^ mobilization, which is necessary for platelet activation. In platelets, raised levels of cAMP result in increased levels of phosphorylated vasodilator-stimulated phosphoprotein (VASP) [26]. Qualitative assessment of VASP phosphorylation was carried out by Western blot. Samples were prepared that had been pretreated with ginkgolide B, incubated with LTA, and were compared with control samples (Figure 5*A*). Levels of phosphorylated VASP, shown by the upper band which represents phosphorylation at residue Ser 157 [26], were increased in both the positive control (prostacyclin treated) and LTA-treated samples indicating a possible role for increased platelet cAMP concentrations in LTA-mediated inhibition. To confirm that this inhibition occurred via PafR, 2 samples were treated with ginkgolide B and one of these with LTA also. In both samples no increase in VASP phosphorylation was observed, demonstrating that VASP phosphorylation resulting from LTA treatment can be blocked by the PafR antagonist. Furthermore, supernatant from *S. aureus* SEJ1 and SEJ1 Δ*gdpP* were able to cause VASP phosphorylation. Supernatant from *S. aureus* SEJ1 Δ*gdpP* Δ*ltaS* was not able to induce phosphorylation, but the effect was restored in supernatant of the complemented strain (Figure 5*A*). These data provide genetic proof of the role of LTA in VASP phosphorylation.

The cAMP concentration in platelet lysates was assayed (Figure 5*B*). A mean *c.* 350% increase of cAMP concentration occurred in platelets incubated with LTA. In platelets pretreated with ginkgolide B, no increase was observed, correlating with the results from the Western blot for VASP phosphorylation. No increase in VASP phosphorylation or cAMP levels was observed in samples treated solely with ginkgolide B.

### LTA Reduces Platelet Thrombus Formation In Vitro and Causes Extended Bleeding Time In Vivo

**Figure 6.**
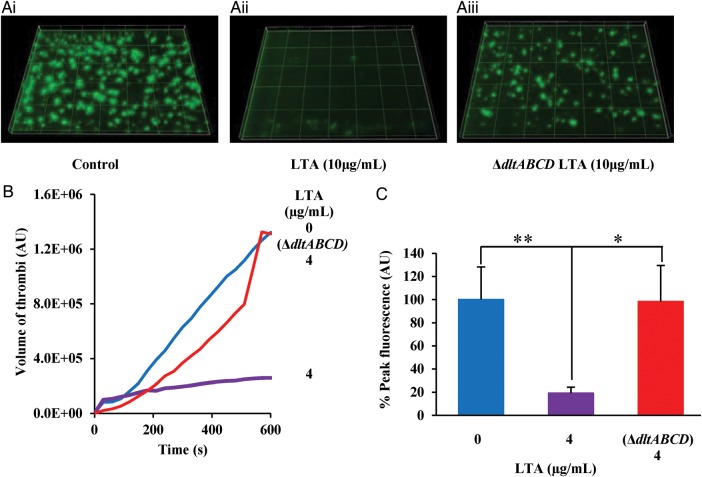
LTA inhibits thrombus formation in vitro. *A*, Platelets within whole human blood were labelled with a lipophilic dye DIOC6. Whole blood was then treated with (*Ai*) tyrodes buffer, (*Aii*) LTA extracted from *Staphylococcus aureus* strains SA113 (10 µg/mL) or (*Aiii*) LTA extracted from *S. aureus* strain SA113 Δ*dltABCD* (10 µg/mL) for 15 minutes. Whole blood was then perfused through collagen coated (400 µg/mL) Vena8Biochip at a flow rate of 20 dynes cm^−2^. Formation of thrombi was recorded using a Z stack capture every 30 seconds for 10 minutes using a Nikon eclipse (TE2000-U) microscope. Thrombus fluorescence intensity was calculated using Slidebook 5 software. *B*, Data represents mean of thrombi volume over the experiment duration. *C*, Data represent mean ± SEM of peak fluorescence intensity. *P < .05. **P < .01. Abbreviations: LTA, lipoteichoic acid; SEM, standard error of the mean.

In order to investigate whether LTA could inhibit thrombus formation in whole blood and under arterial flow conditions, whole human blood was perfused through collagen coated biochips in the presence of tyrodes buffer and LTA from wild-type *S. aureus* or from *S. aureus* SA113 Δ*dltABCD* (Figure 6*A*). In blood pretreated with LTA, a significantly reduced thrombus size, compared to the vehicle treated control, was observed (*P* < .001; Figure 6*A* and 6*B*). Additionally peak fluorescence was reduced by approximately 85% (Figure 6*C*). Blood treated with LTA extracted from SA113 Δ*dltABCD* showed a slower rate of thrombus formation however at the end of the 10 minutes the thrombi present were no different in size than vehicle treated (*P* > .05; Figure 6*B*). The mean peak fluorescence of thrombi formed in the presence of LTA from *S. aureus* Δ*dltABCD* showed no difference to that of the control but was significantly different than wild-type LTA treated (*P* < .05; Figure 6*C*). Inhibition of thrombus formation in blood was consistent with the reduced platelet function observed in washed platelets (Figure 1*Ai*–*Eii*).

**Figure 7.**
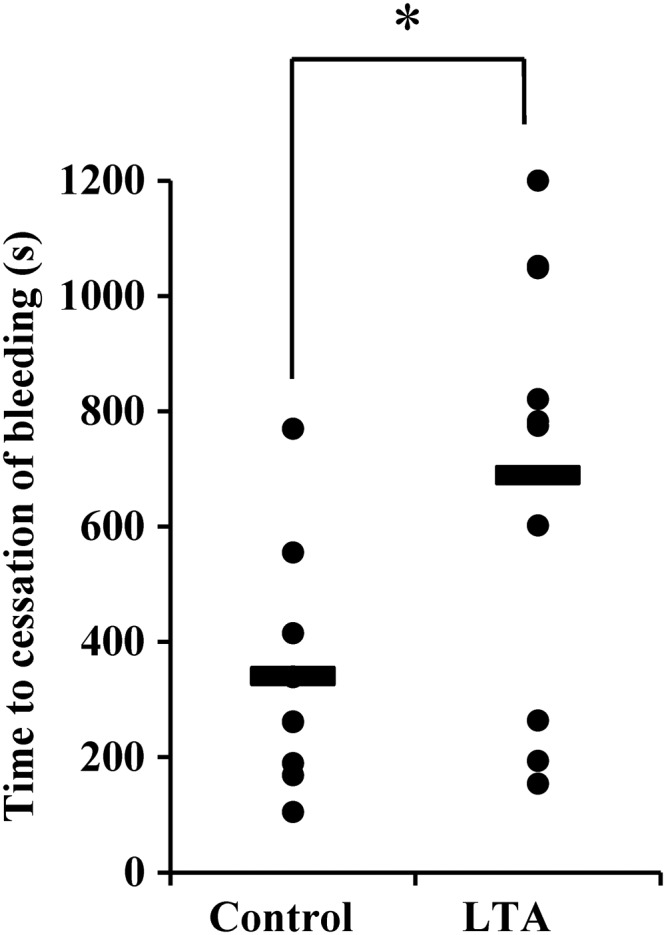
LTA affects hemostasis in vivo. LTA (10 µg/mL) (n = 11) or PBS (n = 11) was administered intravenously to mice and time to cessation of bleeding following a tail biopsy was measured. Data represent individual mice and horizontal lines refer to mean values of seconds until cessation of bleeding. *P < .01. Abbreviations: LTA, lipoteichoic acid; PBS, phosphate-buffered saline.

To substantiate these findings, an in vivo mouse model of bleeding was used. Previous reports have proposed that mouse platelets may lack PafR [27]; however, by Western blotting, we identified a *c.* 48 kDa band in mouse platelets that comigrates with PafR on human platelets (Supplementary Figure 3*A*). Similarly, Paf was a weak agonist, compared to collagen, for mouse and human platelet activation (Supplementary Figure 3*B*) and LTA inhibited platelet activation by collagen in mouse platelets (Supplementary Figure 3*C*). The effect of LTA on maintenance of hemostasis was measured by a tail bleed assay. Infusion of *S. aureus* LTA into rodents does not induce shock or affect blood pressure [28]. The mean bleeding time of vehicle-treated (PBS) mice was 340 seconds following tail biopsy (Figure 7). In LTA-treated mice mean time to cessation of bleeding increased significantly (*P* < .01), more than doubling to 690 seconds.

LTA has multiple roles on many different host cell types, and we cannot rule out an additional role for the endothelium in the tail bleeding experiments. But by measuring thrombus formation in the absence of endothelial cells, we have confirmed a role for LTA alteration of platelet function in thrombus formation. Taken together, these data demonstrate that LTA has a role in the inhibition of thrombosis and hemostasis.

## DISCUSSION

The interplay between *S. aureus* and its human host is complex. Numerous host and pathogen factors are involved in the interaction, and many have multiple activities. *S. aureus* LTA is a polymeric glycerol-phosphate molecule that can be fixed to the cell membrane by a lipid anchor and has a well-established role in several host-pathogen interactions. Although attached to the cell envelope, LTA is also released into the *S. aureus* supernatant, a process accelerated by some antibiotics [29]. In vivo during *S. aureus* infections, LTA has been detected, albeit by a different method to ours, at up to 10 µg/mL [30]. *S. aureus* modifies LTA with D-alanine, which confers multiple effects on its function. Interestingly, the presence of D-alanine on *S. aureus* teichoic acids confers resistance to platelet microbicidal protein, a product of activated platelets [31]. We have shown that D-alanylation increases the platelet-inhibitory potency of LTA. Thus the *dltABCD* operon plays a dual role in protecting *S. aureus* against the bactericidal effects of platelet activation.

Interestingly, LTA from wild-type *S. aureus* was more inhibitory than that from the isogenic *dltABCD* mutant. However, there was no difference in the ability of *S. aureus* cells to induce platelet activation in the absence of exogenous LTA. Both D-alanine residues and lipid anchor have been reported to be required for stimulation of cytokine production in human whole blood and mouse monocytes [32]. This may explain our observation, as the lipid, which is usually embedded within the bacterial membrane, would only be exposed to host cell receptors upon release of LTA. Alternatively, measurements of the *S. aureus* cell wall show it to be sufficiently thick that the commonly depicted schematic, in which LTA chains extend through the cell wall and are exposed on the surface, may be incorrect [33].

Many of the ascribed functions of LTA have been determined using commercial preparations [24, 34], which were subsequently shown to contain contaminants responsible for the activity [10, 18, 35]. Thus results obtained using such preparations may remain open to question. We confirmed that properly purified LTA does inhibit platelet activation. Moreover, LTA inhibits activation of platelets by multiple physiological agonists and whole *S. aureus* cells, each of which trigger activation in different ways, suggesting that LTA blocks a common downstream effect. Ca^2+^ mobilization, a critical stage in platelet activation, was inhibited and is accompanied by increased cellular cAMP concentrations. LTA interacts with PafR, a cell surface receptor that couples to different G proteins to activate cellular responses that differ between cell types [36–40]. In platelets, the G-proteins that interact with PafR remain to be determined. However, PafR does not signal through the pertussis toxin-sensitive G_i_ and G_o_ proteins in platelets [41]. It is well documented that platelet GPCRs can influence cellular cAMP concentrations, thereby inhibiting Ca^2+^ flux [42]. In other cells, PafR interacts with multiple G proteins, resulting in activation of distinct signaling pathways. Indeed, differential PafR signaling in response to agonists and inverse agonists has been reported [43]. Leukocyte responses to Paf utilize pertussis toxin-insensitive and -sensitive G protein(s) [44, 45]. In CHO cells, Paf activation of p38 MAPK occurs through G_q_ protein, but Paf activation of extracellular signal-regulated kinases 1 and 2 occurs via signaling through G_o_ protein [46]. PafR signaling in HUVECs, including cAMP production by stimulation of adenylate cyclase, occurs via the G_q_ protein [47]. The exact nature of the signaling events that lead to the increased platelet cAMP levels will be the topic of future pharmacological studies.

The ability of *S. aureus* to induce platelet activation is well documented [3] and is presumed to be important in the development of bacterial endocarditis. *S. aureus* therefore possesses the ability both to positively and to negatively influence thrombus formation. It seems questionable that platelet activation is advantageous for any bacteria. In doing so, a pathogen becomes enmeshed in a thrombus that can lead to the death of the host, leaving the bacterium unable to continue the infectious cycle. Indeed, the ability to inhibit platelet activation presumably confers an advantage to pathogens during infection. Because they are rich sources of bioactive molecules, some of which are bactericidal, platelets have roles in modulating other cellular functions, including those of the innate and acquired immune systems [48] and as such serve to alert the host to the presence of an infection. Activated platelets can engulf *S. aureus*, although whether this occurs in vivo or has any role in infection remains to be determined [49]. Furthermore, direct interaction with activated platelets induces hyperactivation of neutrophils, enhancing their already potent antibacterial activity [50].

## Supplementary Data

Supplementary materials are available at *The Journal of Infectious Diseases* online (http://jid.oxfordjournals.org/). Supplementary materials consist of data provided by the author that are published to benefit the reader. The posted materials are not copyedited. The contents of all supplementary data are the sole responsibility of the authors. Questions or messages regarding errors should be addressed to the author.

Supplementary Data

## References

[1] Yeaman MR, Sullam PM, Dazin PF, Norman DC, Bayer AS (1992). Characterization of *Staphylococcus aureus*-platelet binding by quantitative flow cytometric analysis. J Infect Dis.

[2] Miajlovic H, Zapotoczna M, Geoghegan JA, Kerrigan SW, Speziale P, Foster TJ (2010). Direct interaction of iron-regulated surface determinant IsdB of *Staphylococcus aureus* with the GPIIb/IIIa receptor on platelets. Microbiology.

[3] Fitzgerald JR, Foster TJ, Cox D (2006). The interaction of bacterial pathogens with platelets. Nat Rev Microbiol.

[4] Madsen SM, Westh H, Danielsen L, Rosdahl VT (1996). Bacterial colonization and healing of venous leg ulcers. Acta Path Microbiol Immunol Scand.

[5] Shannon O, Uekötter A, Flock J-I (2005). Extracellular fibrinogen binding protein, Efb, from *Staphylococcus aureus* as an antiplatelet agent *i*n vivo. Thromb Haemost.

[6] Zhang X, Liu Y, Gao Y (2011). Inhibiting platelets aggregation could aggravate the acute infection caused by *Staphylococcus aureus*. Platelets.

[7] Sheu JR, Lee CR, Lin CH (2000). Mechanisms involved in the antiplatelet activity of *Staphylococcus aureus* lipoteichoic acid in human platelets. Thromb Haemost.

[8] Peschel A, Otto M, Jack RW, Kalbacher H, Jung G, Gotz F (1999). Inactivation of the *dlt* operon in *Staphylococcus aureus* confers sensitivity to defensins, protegrins, and other antimicrobial peptides. J Biol Chem.

[9] Weidenmaier C, Kokai-Kun JF, Kristian SA (2004). Role of teichoic acids in *Staphylococcus aureus* nasal colonization, a major risk factor in nosocomial infections. Nat Med.

[10] Stoll H, Dengjel J, Nerz C, Gotz F (2005). *Staphylococcus aureus* deficient in lipidation of prelipoproteins is attenuated in growth and immune activation. Infect Immun.

[11] Corrigan RM, Abbott JC, Burhenne H, Kaever V, Gründling A (2011). c-di-AMP is a new second messenger in *Staphylococcus aureus* with a role in controlling cell size and envelope stress. PLoS Pathog.

[12] Ames BN (1966). Assay of inorganic phosphate, total phosphate and phosphatases. Methods Enzymol.

[13] Moraes LA, Spyridon M, Kaiser WJ (2010). Non-genomic effects of PPARγ ligands: inhibition of GPVI-stimulated platelet activation. J Thromb Haemost.

[14] Fedtke I, Mader D, Kohler T (2007). A *Staphylococcus aureus ypfP* mutant with strongly reduced lipoteichoic acid (LTA) content: LTA governs bacterial surface properties and autolysin activity. Mol Microbiol.

[15] Vinogradov E, Sadovskaya I, Li J, Jabbouri S (2006). Structural elucidation of the extracellular and cell-wall teichoic acids of *Staphylococcus aureus* MN8 m, a biofilm forming strain. Carbohydr Res.

[16] Siboo IR, Cheung AL, Bayer AS, Sullam PM (2001). Clumping factor A mediates binding of *Staphylococcus aureus* to human platelets. Infect Immun.

[17] Que Y-A, Haefliger J-A, Piroth L (2005). Fibrinogen and fibronectin binding cooperate for valve infection and invasion in *Staphylococcus aureus* experimental endocarditis. J Exper Med.

[18] Hashimoto M, Tawaratsumida K, Kariya H (2006). Not lipoteichoic acid but lipoproteins appear to be the dominant immunobiologically active compounds in *Staphylococcus aureus*. J Immunol.

[19] Iwasaki H, Shimada A, Yokoyama K, Ito E (1989). Structure and glycosylation of lipoteichoic acids in *Bacillus* strains. J Bacteriol.

[20] Fischer W, Behr T, Hartmann R, Peter-Katalini J, Egge H (1993). Teichoic acid and lipoteichoic acid of *Streptococcus pneumoniae* possess identical chain structures. Euro J Biochem.

[21] Lotz S, Aga E, Wilde I (2004). Highly purified lipoteichoic acid activates neutrophil granulocytes and delays their spontaneous apoptosis via CD14 and TLR2. J Leukoc Biol.

[22] Draing C, Sigel S, Deininger S (2008). Cytokine induction by Gram-positive bacteria. Immunobiology.

[23] Schröder NWJ, Morath S, Alexander C (2003). Lipoteichoic acid (LTA) of *Streptococcus pneumoniae* and *Staphylococcus aureus* activates immune cells via Toll-like receptor (TLR)-2, lipopolysaccharide-binding protein (LBP), and CD14, whereas TLR-4 and MD-2 are not involved. J Biol Chem.

[24] Zhang Q, Mousdicas N, Yi Q (2005). Staphylococcal lipoteichoic acid inhibits delayed-type hypersensitivity reactions via the platelet-activating factor receptor. J Clin Invest.

[25] Vogensen SB, Strømgaard K, Shindou H (2003). Preparation of 7-substituted ginkgolide derivatives: potent platelet activating factor (PAF) receptor antagonists. J Med Chem.

[26] Li Z, Ajdic J, Eigenthaler M, Du X (2003). A predominant role for cAMP-dependent protein kinase in the cGMP-induced phosphorylation of vasodilator-stimulated phosphoprotein and platelet inhibition in humans. Blood.

[27] Rowley JW, Oler A, Tolley ND (2011). Genome wide RNA-seq analysis of human and mouse platelet transcriptomes. Blood.

[28] Kengatharan KM, De Kimpe S, Robson C, Foster SJ, Thiemermann C (1998). Mechanism of Gram-positive shock: identification of peptidoglycan and lipoteichoic acid moieties essential in the induction of nitric oxide synthase, shock, and multiple organ failure. J Exp Med.

[29] van Langevelde P, van Dissel JT, Ravensbergen E, Appelmelk BJ, Schrijver IA, Groeneveld PH (1998). Antibiotic-induced release of lipoteichoic acid and peptidoglycan from *Staphylococcus aureus*: quantitative measurements and biological reactivities. Antimicrob Agents Chemother.

[30] Travers JB, Kozman A, Mousdicas N (2010). Infected atopic dermatitis lesions contain pharmacologic amounts of lipoteichoic acid. J Allergy Clin Immunol.

[31] Weidenmaier C, Peschel A, Kempf VA, Lucindo N, Yeaman MR, Bayer AS (2005). DltABCD- and MprF-mediated cell envelope modifications of *Staphylococcus aureus* confer resistance to platelet microbicidal proteins and contribute to virulence in a rabbit endocarditis model. Infect Immun.

[32] Morath S, Stadelmaier A, Geyer A, Schmidt RR, Hartung T (2002). Synthetic lipoteichoic acid from *Staphylococcus aureus* is a potent stimulus of cytokine release. J Exper Med.

[33] Reichmann NT, Gründling A (2011). Location, synthesis and function of glycolipids and polyglycerolphosphate lipoteichoic acid in Gram-positive bacteria of the phylum *Firmicutes*. FEMS Microbiol Lett.

[34] Voorhees T, Chang J, Yao Y, Kaplan MH, Chang CH, Travers JB (2011). Dendritic cells produce inflammatory cytokines in response to bacterial products from *Staphylococcus aureus*-infected atopic dermatitis lesions. Cell Immunol.

[35] Schmaler M, Jann NJ, Ferracin F (2009). Lipoproteins in *Staphylococcus aureus* mediate inflammation by TLR2 and iron-dependent growth in vivo. J Immunol.

[36] Murphy S, Welk G (1990). Hydrolysis of polyphosphoinositides in astrocytes by platelet-activating factor. Eur J Pharmacol.

[37] Ye RD, Prossnitz ER, Zou AH, Cochrane CG (1991). Characterization of a human cDNA that encodes a functional receptor for platelet activating factor. Biochem Biophys Res Commun.

[38] Yue TL, Stadel JM, Sarau HM (1992). Platelet-activating factor stimulates phosphoinositide turnover in neurohybrid NCB-20 cells: involvement of pertussis toxin-sensitive guanine nucleotide-binding proteins and inhibition by protein kinase C. Mol Pharmacol.

[39] Amatruda TT, Gerard NP, Gerard C, Simon MI (1993). Specific interactions of chemoattractant factor receptors with G-proteins. J Biol Chem.

[40] Mazer BD, Sawami H, Tordai A, Gelfand EW (1992). Platelet-activating factor-mediated transmembrane signaling in human B lymphocytes is regulated through a pertussis- and cholera toxin-sensitive pathway. J Clin Invest.

[41] Hwang SB (1988). Identification of a second putative receptor of platelet-activating factor from human polymorphonuclear leukocytes. J Biol Chem.

[42] Schwarz UR, Walter U, Eigenthaler M (2001). Taming platelets with cyclic nucleotides. Biochem Pharmacol.

[43] Dupré DJ, Thompson C, Chen Z (2007). Inverse agonist-induced signaling and down-regulation of the platelet-activating factor receptor. Cell Signal.

[44] Haribabu B, Zhelev DV, Pridgen BC, Richardson RM, Ali H, Snyderman R (1999). Chemoattractant receptors activate distinct pathways for chemotaxis and secretion: role of G-protein usage. J Biol Chem.

[45] Brown SL, Jala VR, Raghuwanshi SK, Nasser MW, Haribabu B, Richardson RM (2006). Activation and regulation of platelet-activating factor receptor: role of G(i) and G(q) in receptor-mediated chemotactic, cytotoxic, and cross-regulatory signals. J Immunol.

[46] Nick JA, Avdi NJ, Young SK (1997). Common and distinct intracellular signaling pathways in human neutrophils utilized by platelet activating factor and FMLP. J Clin Invest.

[47] Deo DD, Bazan NG, Hunt JD (2004). Activation of platelet-activating factor receptor-coupled G alpha q leads to stimulation of Src and focal adhesion kinase via two separate pathways in human umbilical vein endothelial cells. J Biol Chem.

[48] Smyth SS, McEver RP, Weyrich AS (2009). Platelet functions beyond hemostasis. J Thromb Haemost.

[49] Youssefian T, Drouin A, Masse JM, Guichard J, Cramer EM (2002). Host defense role of platelets: engulfment of HIV and *Staphylococcus aureus* occurs in a specific subcellular compartment and is enhanced by platelet activation. Blood.

[50] Clark SR, Ma AC, Tavener SA (2007). Platelet TLR4 activates neutrophil extracellular traps to ensnare bacteria in septic blood. Nat Med.

