# Supplementary material for: Staphylococcus aureus Lipoteichoic Acid Inhibits Platelet Activation and Thrombus Formation via the Paf Receptor
Source: J Infect Dis. 2013 Aug 2;208(12):2046–57. doi: 10.1093/infdis/jit398 (PMC3836464; doi:10.1093/infdis/jit398)
Supplement: Supplementary Data [file supp_208_12_2046__index.html]

Staphylococcus aureus Lipoteichoic Acid Inhibits Platelet Activation and Thrombus Formation via the Paf Receptor — Staphylococcus aureus Lipoteichoic Acid Inhibits Platelet Activation and Thrombus Formation via the Paf Receptor — Supplementary Data 

# *Staphylococcus aureus* Lipoteichoic Acid Inhibits Platelet Activation and Thrombus Formation via the Paf Receptor

## Supplementary Data

Supplementary Data

**Files in this Data Supplement:**

- Supplementary Data - Doc file
- Supplementary Figure 1 - tiff file
- Supplementary Figure 2 - tiff file
- Supplementary Figure 3 - tiff file
