# Supplementary material for: Staphylococcus aureus Lipoteichoic Acid Inhibits Platelet Activation and Thrombus Formation via the Paf Receptor
Source: J Infect Dis. 2013 Aug 2;208(12):2046–57. doi: 10.1093/infdis/jit398 (PMC3836464; doi:10.1093/infdis/jit398)
Supplement: Supplementary Data [file supp_jit398_jit398supp.doc]

**Figure S1**

**LTA extracted from cells is as inhibitory as LTA extracted from supernatant and permanently inhibits platelets.** (A) Washed human platelets (4 x 10^8^ cells mL^-1^) were pre-incubated with LTA extracted from *S. aureus* supernatant or cells, or tyrodes buffer and stimulated with CRP-XL (0.5μg mL^-1^). Aggregation was measured for 90 s. Data are plotted as percentage inhibition of aggregation (vehicle treated representing 100% aggregation) and represent mean values + SEM. *P>0.05. (B) A representative aggregation trace of washed human platelets (4 x 10^8^ cells mL^-1^) were pre-incubated with LTA purified from *S. aureus* SA113 at several concentrations or tyrodes buffer for 15 minutes before stimulation with thrombin (0.05units mL^-1^). Aggregation was measured as change in light transmission for 300 s.

**Figure S2**

**LTA extracted from SA113 Δ*tagO* and Δ*lgt* strains is as inhibitory as LTA extracted from the parental wild-type.** Washed platelets were incubated for 15 minutes with LTA purified from *S. aureus* SA113*,* SA113 Δ*tagO* or SA113 Δ*lgt*, followed by stimulation with CRP-XL. (A) Representative aggregation traces of washed platelets. Aggregation was measured for 90 s. (B) Data are plotted as percentage inhibition of aggregation and represent mean values + SEM. *P>0.05

**Figure S3**

**LTA inhibits mouse platelets.** (A) Washed human platelet (4 x 10^8^ cells mL^-1^) or washed mouse platelet (4 x 10^8^ cells mL^-1^) lysates were immune-blotted with an anti-PafR antibody. **(**B) Respresentative aggregation trace of washed mouse platelets (2.6 x 10^8^ cells mL^-1^) were pre-incubated with LTA purified from *S. aureus* SA113 (10µg mL^-1^) or tyrodes buffer for 15 minutes before stimulation with collagen (2.5µg mL^-1^). Aggregation was measured as change in light transmission for 90 s. Data are plotted as percentage inhibition of aggregation (vehicle treated representing 100% aggregation) and represent mean values ± SEM. (C) Washed mouse platelets (2.6 x 10^8^ cells mL^-1^) or washed human platelest (4 x 10^8^ cells mL^-1^) were stimulated with either platelet activating factor (40µg mL^-1^) or collagen (2.5µg mL^-1^). Aggregation was measured as change in light transmission for 90s.
