# Supplementary figures and images for: Staphylococcus aureus Lipoteichoic Acid Inhibits Platelet Activation and Thrombus Formation via the Paf Receptor
Source: J Infect Dis. 2013 Aug 2;208(12):2046–57. doi: 10.1093/infdis/jit398 (PMC3836464; doi:10.1093/infdis/jit398)

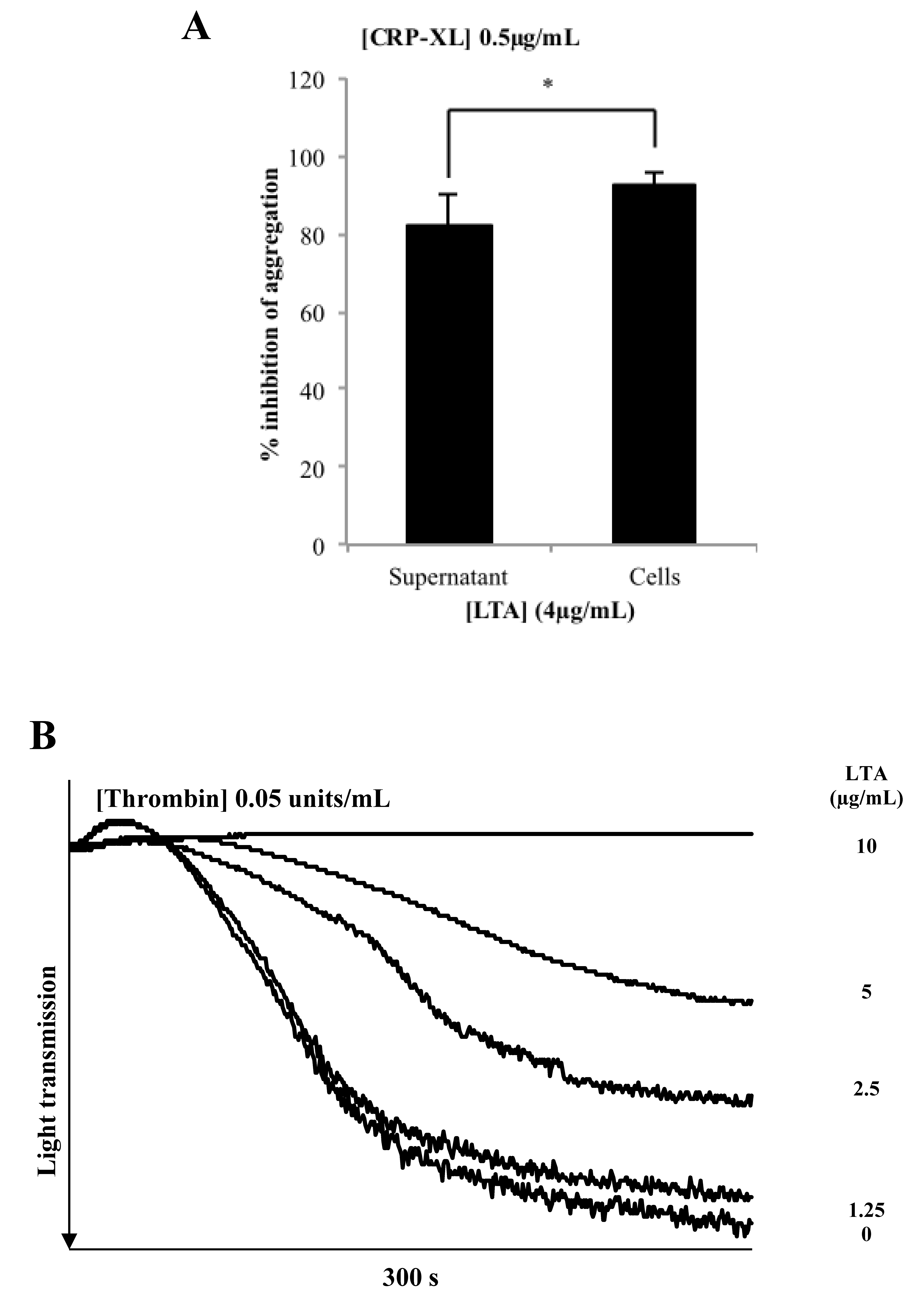

Supplement: Supplementary Data [file supp_jit398_jit398supp_fig1.tif]

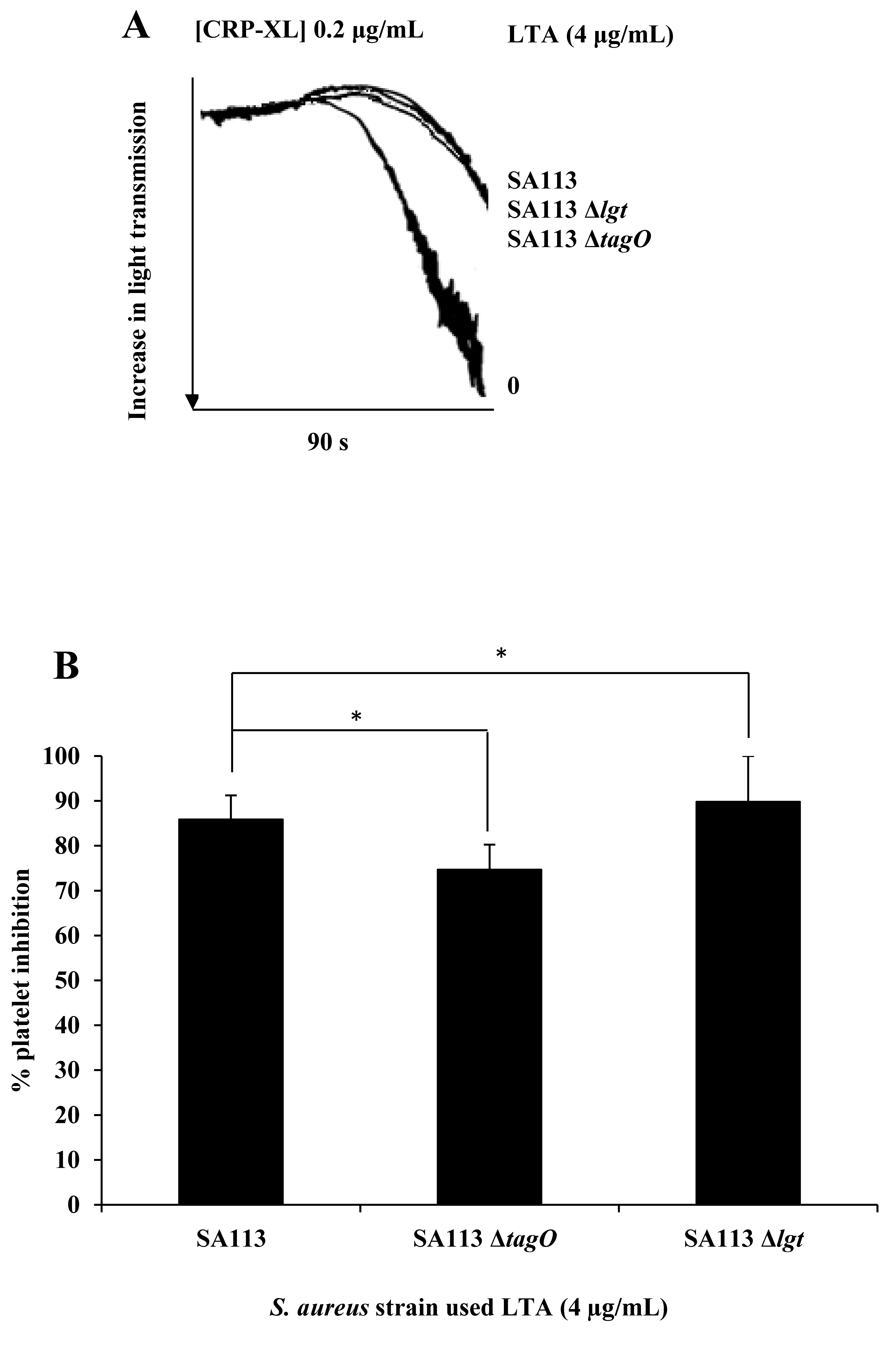

Supplement: Supplementary Data [file supp_jit398_jit398supp_fig2.tif]

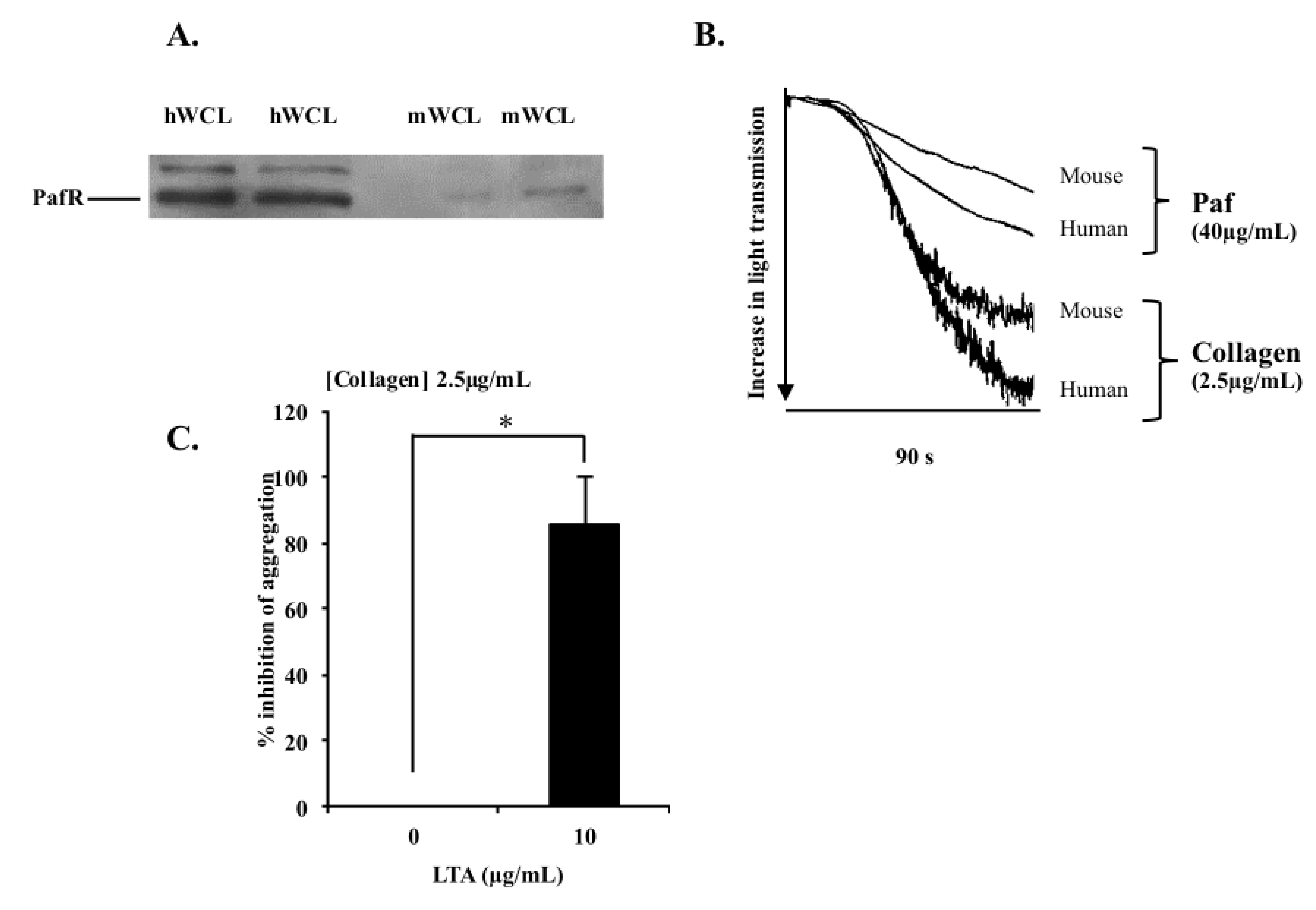

Supplement: Supplementary Data [file supp_jit398_jit398supp_fig3.tif]
